# Examining Patient- and Community-Level Factors Associated with Pediatric Mental Healthcare Access Within a Patient Navigation Program

**DOI:** 10.1007/s10597-024-01258-7

**Published:** 2024-03-20

**Authors:** Caitlin Koob, Mackenzie Stuenkel, Ryan J. Gagnon, Sarah F. Griffin, Kerry Sease

**Affiliations:** 1 Department of Public Health Sciences, Clemson University, 501 Edwards Hall, Clemson, SC 29634 USA; 2 Prisma Health Children’s Hospital-Upstate, Greenville, SC USA; 3 Department of Parks, Recreation, Tourism, and Management, Clemson University, Clemson, SC USA; 4 University of South Carolina School of Medicine Greenville, Greenville, SC USA

**Keywords:** Pediatric mental health, Systems navigation, Social drivers of health, Community health, Health equity

## Abstract

In 2021, national leaders in the United States declared a “national youth mental health crisis.” Still, only 1-in-4 children receive adequate mental healthcare access. Patient Navigator Programs (PNPs) can improve children’s referral-to-connection to mental health services. We examined patient- and community-level factors associated with pediatric mental healthcare access. Pediatric Support Services (PSS) is a PNP that triages mental and behavioral health referrals within a large health system in a southeastern state. This study analyzes PSS data from September 2017-March 2023 and Child Opportunity Index 2.0 state-normed zip-code level data to assess social drivers of health estimates. Structural equation modeling was conducted between patient- and community-level factors and connection to mental health services. Overall, 62.7% of children connected to mental health services since PSS’ inception. Regardless of SDOH, as children get older, they are more likely to connect with mental health services (β = .053, *SE* = .010, *p* < .001). Children with greater number of referral needs are more likely to connect with mental health services (β = .034, *SE* = .011, *p* = .002). Further, children who live in communities with higher opportunity levels are more likely to connect with mental health services (β = .016, *SE* = .008, *p* = .040), suggesting that children who live in low-income communities experience more barriers to mental healthcare. Social drivers may inform referral practices and tiered navigation support for optimal mental healthcare access among children. Further research should demonstrate the effectiveness of PNPs integrated within healthcare and community-based settings.

## Introduction

Approximately 13% of children worldwide are estimated to have a mental health condition at any given time, with nearly 75% of conditions developing before age 18 and only 25% receiving adequate specialist care (Anderson et al., 2014; Barican et al., 2022; Lui et al., 2022). In 2021, national leaders in the United States (US) declared a “national youth mental health crisis,” citing worsened mental health prevalence and symptomology since the onset of the COVID-19 pandemic (Jones et al., 2021; Tolliver & Hostutler, 2022). Existing research suggests that youth experienced increased anxiety and depression symptoms and decreased overall life satisfaction within the early months of COVID-19, and the long-term effects of the pandemic on youth mental health remain unknown (Jones et al., 2021; Magson et al., 2021; Power et al., 2020; Rajmil et al., 2021). Despite the significant and recently exacerbated need, there is a significant gap in access to mental health care services in the US and associated barriers for specific pediatric patient populations, such as children with food and/or housing insecurity (Barican et al., 2022; Burrell et al., 2023; Lui et al., 2022; Nayak et al., 2022; Rajmil et al., 2021). Prevention and early treatment of mental health services in the developmental phases of children is critical to long-term health, further emphasizing the need for interventions that address this care gap (Fusar-Poli, 2019).

Additionally, social drivers of health (SDOH), such as chronic food insecurity or unstable housing, are known to influence longitudinal mental health outcomes (Garg et al., 2016). South Carolina (SC) demonstrates a disproportionately high rate of food insecurity among children and their families, negatively impacting longitudinal health outcomes and often causing chronic stress across critical developmental periods (Burke et al., 2018; Drucker et al., 2019; Dush, 2020; Tester et al., 2020). Further, children who live in low-income households are increasingly more likely to developing mental health conditions and have ongoing unmet mental health needs that will persist across the lifespan (Burke et al., 2018; Hodgkinson et al., 2017; Nayak et al., 2022). Still, SDOH factors are often excluded from clinical data and indicate a missed opportunity to inform patient care. Existing research suggests that referral practices without follow-up support to ensure service connection is debatably unethical (Garg et al., 2016). The Child Opportunity Index 2.0 (COI 2.0) is a validated measurement tool that estimates a child’s zip-code level, socioeconomic, environmental, and educational conditions and rates their “opportunity” as a contributing factor of long-term health (Anderson et al., 2014; Garg & Dworkin, 2016). COI 2.0 is founded on the Life Course Perspective, where environmental factors in a child’s upbringing affect development and influence health outcomes across the lifespan (Bengtson & Allen, 1993; Johnson et al., 2011). With knowledge of patients’ SDOH-related risk, primary care providers are uniquely positioned to help children and their families navigate social drivers that may increase their risk of experiencing mental health conditions and inadequate mental health care (Deferio et al., 2019; Hodgkinson et al., 2017). Paired with early detection and treatment, it is critical to consider socio-demographic factors associated with increased risk of developing mental health conditions among children and identify interventions to overcome service-level barriers, including waitlists, inflexible service times, financial cost, availability of mental health specialists, and lack of knowledge regarding available services (Anderson et al., 2014; Fusar-Poli, 2019; Matandika et al., 2022). Still, effective interventions—that are conscious of patients’ circumstances related to SDOH—are a critical piece of supporting a continuum of care from referral to service connection (Dworkin & Garg, 2019; Garg & Dworkin, 2016; Garg et al., 2016).

Patient navigation programs (PNP) are a potential solution to the mental health care gap with demonstrated effectiveness in increasing access to services among children and their families, particularly among models with collaborative components between navigators and clinical providers (Bowles et al., 2020; Gunn et al., 2017). A majority of PNPs target adult populations and are designed to meet highly variable, specific patient populations (Carter et al., 2018; Gunn et al., 2017; Ustjanauskas et al., 2016). Still, PNPs among pediatric populations demonstrate significantly improved referral-to-service connection rates and demonstrate the potential to improve developmental outcomes with early detection and treatment of mental and behavioral health needs (Colizzi et al., 2020; Conroy et al., 2018). PNPs offer patient and family support to navigate the dynamic health system, emerging as a strategy to reduce barriers to care, fragmented health service delivery, and ongoing unmet health needs (Bowles et al., 2020; Carter et al., 2018; Fusar-Poli, 2019; Hodgkinson et al., 2017). SDOH data is rarely collected prospectively within these models and is not typically included in clinical research to provide context for patients’ prospective long-term health outcomes, making it difficult to monitor longitudinal service use patterns (Deferio et al., 2019; Reid et al., 2019). There is a great need to integrate SDOH factors into pediatric care settings to avoid referrals with overwhelming barriers to care, including cost, primary language, type of services, and family structure, that are not realistic for patients and their families and to ensure accessible healthcare services over time (Bettenhausen et al., 2022; Burrell et al., 2023; Deferio et al., 2019; Lui et al., 2022; Reid et al., 2019). The COI 2.0 provides an opportunity to contextualize patient needs and is a tool for providers’ to address their patients’ needs (Anderson et al., 2014).

Pediatric Support Services (PSS) is a novel, active PNP that is integrated into primary care clinics and triages referrals for mental and behavioral health concerns and for SDOH concerns, within a large health system in South Carolina (SC), USA. Previous research in SC suggests some children may be at-risk of experiencing ongoing unmet mental health needs based on patient demographics, neighborhood factors, and geographic factors such as rurality—suggesting the role of integrated social work perspectives as a solution for equitable healthcare access and experiences (Addy et al., 2015; Babatunde et al., 2021; DeWitt et al., 2020; McElligott & Summer, 2013). This study merges COI 2.0 with data from PSS to inform navigator protocols and practices for tailored support of children who are referred to mental health services within this health system. Further research is needed to examine multi-level factors that influence referral-to-service connection among patients with mental health needs (Burrell et al., 2023; Council on children with disabilities and medical home implementation project advisory committee et al., 2014; Lui et al., 2022). PSS was designed, within this health system in SC, as an equitable, novel approach that (1) integrates care between the health system and community and (2) addresses the mental health needs of children and their families, with active support from trained navigators to facilitate referral-to-service connection. With demonstrated effectiveness, the PSS model may be implemented in health systems statewide and may be considered for replication in other regions nationwide. The purpose of this study is to understand individual- and community-level factors that may influence children’s connection to mental health services within the context of PSS in SC, USA. Therefore, this study aims to:

1. Examine the impact of patient- and community-level factors on connection to pediatric mental health services within an active PNP; and,
2. Examine the impact of social drivers on connection to pediatric mental health services within an active PNP.

## Methods

### Study Design, Setting, and Population

In this retrospective study, we analyzed data nested within a larger, ongoing evaluation of PSS. PSS is operated within a children’s hospital in Upstate, SC and supports patient referral to service connection from a network of 15 primary care clinics. This health system is the largest not-for-profit health organization in the state, operating as an academic-clinical institution, with academic and community-based partnerships statewide. With a service area spanning over several counties, the health system sees a diverse population from rural and urban settings.

### Intervention

**Fig. 1:**
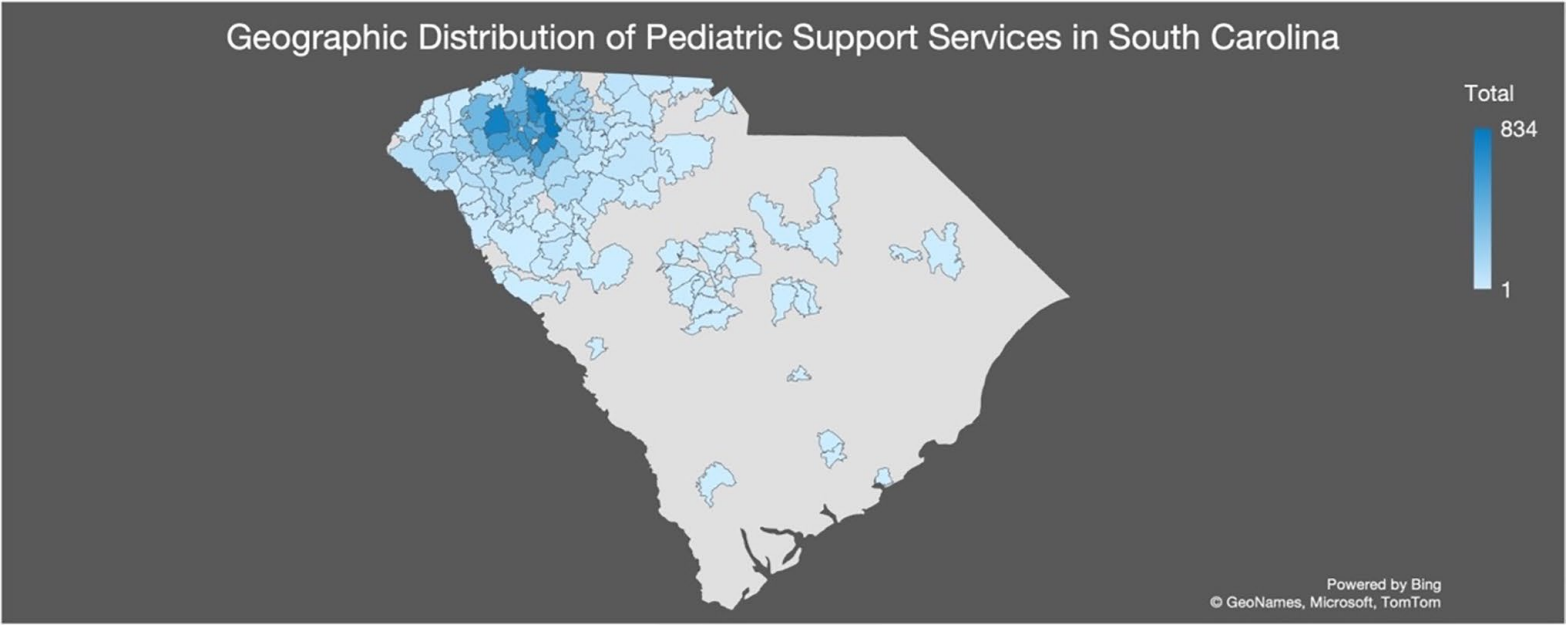
Density of geographic distribution of Pediatric Support Services’ reach in South Carolina by patient-level zip code of residence

PSS was implemented systemwide in 2017 and is currently integrated within 15 pediatric primary care clinics in the Upstate, triaging over 17,000 referrals to date. The density of the geographic distribution of patients reached by PSS is illustrated in Fig. 1. Children are eligible to be referred to PSS by primary care providers for additional support due to mental, behavioral, and developmental concerns, food and housing assistance, or legal support. PSS triages and navigates referrals from primary care providers to facilitate referral-to-service connection. Navigators are social workers, with extensive knowledge on existing services and identifying and addressing patients’ needs, connecting children and their families with appropriate system and community-based services. Once referred, navigators make initial contact with patients’ parent/legal guardian for service connection, collect demographic information, identify specific patient needs, and refer them to available system and community-based services. Services include health system mental, behavioral, and developmental health specialists, social workers, health literacy and education programs, food assistance programs (both community-based and federal), home visiting and parenting support programs, and culturally responsive support organizations. Navigators then follow-up after one month from initial contact to optimize referral-to-service connection and identify accessibility barriers to be addressed.

### Data Sources

Navigators access patient medical charts from referral, utilize an internal resource database to search for relevant services (detailed above), and document patient outreach information in a Research Electronic Data Capture (REDCap) database—a HIPAA-compliant, electronic database that securely stores patient information for translational research (Harris et al., 2009). This study utilizes data housed in REDCap that merges outreach data with electronic health records (EPIC) (Harris et al., 2009; Shalhout et al., 2022). This analysis includes a subset of data since PSS’ inception in September 2017 through March 2023 (N = 3930). Patients included in this study were those: (1) were aged 0 to 18, (2) who received at least 1 referral for mental health services, and (3) had follow-up information recorded at 2-months after their initial referral. This study was approved by [Health System Blinded for Review] Institutional Review Board.

### Measures

#### Service Connection

The primary outcome of this study was “connection to mental health services” at time of one month follow-up from date of successful initial contact with navigator, following pediatricians’ referral. “Connection to services” was defined as those who have received or who are pending services via scheduled appointments. Patients are recorded as pending when they have an appointment scheduled within the following one month, with an internal quality improvement project demonstrating nearly 90% completion of appointments for patients recorded as pending. Patients who are pending connection to services are considered “connected” and differentiated from patients on waitlists to represent the inflow of pediatric mental health needs relative to service provider availability within the health system. (Tolliver & Hostutler, 2022). Pending patients can schedule appointments, whereas patients on waitlists suggest an overburdened health system and are thus recorded as “not connected.”

#### The Child Opportunity Index 2.0

Due to the assessment of and triage to services for SDOH within PSS, it is critical to understand the influence of SDOH factors on connection to mental health services for informed PSS adaptations (Deferio et al., 2019; Dworkin & Garg, 2019; Sederer, 2016). In this study, patients’ zip-codes in their electronic health records were matched with COI 2.0 to estimate the impact of SDOH at zip code level (Acevedo-Garcia et al., 2020; Bettenhausen et al., 2022; Figueroa et al., 2020). This study uses 2015 state-normed zip code level estimates of the 29 indicators of COI 2.0 across three domains: Health and Environment, Education, and Social and Economic (Appendix A) (Acevedo-Garcia et al., 2014, 2020; Jutte et al., 2021). Indicators of COI 2.0 include zip code level measures of: access to healthy food, housing vacancy rates (Health and Environment), third grade reading and math proficiency, high school education rate (Education), poverty rate, and public assistance rate (Social and Economic).Child “opportunity” is scored on a 5-point scale, ranging from “very high” to “very low” opportunity with higher scores indicating greater opportunity (Bettenhausen et al., 2022). A child’s “opportunity” score is a method of operationalizing their SDOH and is referred to as their “SDOH position” throughout this paper.

#### Independent Variables and Demographics

Children were characterized by demographic characteristics, including age (categories, in years: 0 to < 3, 3 to 5, 6 to 11, 12 to 16, 17+), biological sex (Male/Female), race and/or ethnicity (non-Hispanic White, non-Hispanic Black, Hispanic, and Other), and primary insurance coverage (Medicaid, Commercial, and Self-Pay/Other). In addition, we hypothesized the number of primary needs at time of referral among patients, and parental mental health as the principal reason for referral may impact a child’s connection to mental health services (Eiser & Varni, 2013; Luby et al., 2013; Ogundele, 2018). Therefore, these variables are included in this analysis.

### Statistical Methods

Descriptive statistics were used to examine patient- and community-level factors and their association(s) with connecting to mental health services among children who were triaged within PSS. Chi-square tests were used to examine differences in connection to mental health services by age, race, sex, primary insurance coverage, and by zip-code level SDOH estimates (Table 1) (Jutte et al., 2021).

**Table 1:** Characteristics of PSS Population by Connection Status (N = 3930)

	Overall		Not connected to services				Connected to Services		*p*
	N	%	N		%		N	%	
	3930	–	1465		37.28		2465	62.72	
Age									< .001
0 to < 3	89	2.43	53		59.55		36	40.45	
3 to 5	670	18.28	319		47.61		351	52.39	
6 to 11	1521	41.49	515		33.86		1006	66.14	
12 to 16	1041	28.40	368		35.35		673	64.65	
17 and older	345	9.41	120		34.78		225	65.22	
Race/ethnicity									.236
Non-Hispanic White	2689	70.34	1020		37.93		1669	62.07	
Non-Hispanic Black	118	3.09	46		38.98		72	61.02	
Hispanic	502	13.13	184		36.65		318	63.35	
Other	514	13.44	171		33.27		343	66.73	
Biological sex									.212
Male	1911	50.86	733		38.36		1178	61.64	
Female	2004	51.19	729		36.37		1275	63.62	
Primary insurance coverage									.250
Medicaid	2125	54.18	775		36.47		1350	63.53	
Commercial	1545	39.39	582		37.67		963	62.33	
Other	252	6.43	105		41.67		147	58.33	
Primary reason for referral									.007
Mental, behavioral, and developmental concerns	2892	73.63	1777		72.12		1115	76.16	
Caregiver/family needs	909	23.14	595		24.15		314	21.45	
High-risk social needs	127	3.23	92		3.73		35	2.39	
Childhood opportunity index (by Domain)									
Health & environment									.134
Very low	658	16.85	245		37.23		413	62.77	
Low	497	12.73	202		40.64		295	59.36	
Moderate	1026	26.27	392		38.21		634	61.79	
High	1291	33.06	475		36.79		816	63.21	
Very high	433	11.09	141		32.56		292	67.44	
Social & economic									.013
Very low	253	6.48	97		38.34		156	61.66	
Low	348	8.91	121		34.77		227	65.23	
Moderate	979	25.07	399		40.76		580	59.24	
High	1071	27.43	412		38.47		659	61.53	
Very high	1254	32.11	426		33.97		828	66.03	
Education									.011
Very low	51	1.31	24		47.06		27	52.94	
Low	401	10.50	131		32.67		270	67.33	
Moderate	977	25.02	386		39.51		591	60.49	
High	1111	28.45	437		39.33		674	60.67	
Very high	1365	34.96	477		34.95		888	65.05	

We addressed our study aims using structural equation modeling (SEM), a latent modeling technique (Sardeshmukh & Vandenberg, 2017). In addition to patient-specific variables, SDOH factors, compartmentalized into three validated domains (Health and Environment, Education, and Social and Economic), were assigned to a SEM to examine their influence on connection to mental health services (Jutte et al., 2021). Due to these factors’ influence on healthcare access, it is critical to consider the role of SDOH within PSS, despite being difficult to directly measure. Therefore, this analysis constructed a latent variable comprised of 29 indicators related to Health and Environment, Education, and Social and Economic domains to represent SDOH. In this analysis, SEM examines the moderating effects of SDOH, a latent variable, between patient-level variables and connection to mental health services. Therefore, SEM is an appropriate analytic technique for this study. This analysis was conducted using the lavaan package within R, version 4.2.3 (Racine, 2012; Rosseel, 2012).

SEM was conducted to identify a “best-fit” model to accurately represent the relations between patient- and community-level factors and connection to mental health services among children triaged through PSS since its inception. This analysis used maximum likelihood estimation to calculate model estimates, which were deemed significant at $a$ = 0.05 (Maydeu-Olivares, 2017). To determine the model of best-fit, the chi-square test statistic and degrees of freedom, root mean squared error of approximation (RMSEA), Tucker-Lewis Index (TLI) and Confirmatory Fit Index (CFI) values were evaluated (Maydeu-Olivares, 2017; Sardeshmukh & Vandenberg, 2017). Established guidelines suggest that TLI and CFI values should be as close to 1 as possible, and RMSEA should be a value of 0.05 or less (Marcoulides et al., 2020; Maydeu-Olivares, 2017; Sardeshmukh & Vandenberg, 2017).

#### A Priori Hypotheses for Structural Equation Model Relationships

Existing literature illustrates the relation between SDOH and mental health, identifying the need to implement validated measurement tools and consider the clinical implications of SDOH (Deferio et al., 2019; Hodgkinson et al., 2017; Matandika et al., 2022). Because PSS triages referrals for SDOH from universal screening in primary care, we hypothesized that zip-code level SDOH estimates—operationalized through COI 2.0—would be significant positive predictors of connection to mental health services (Bettenhausen et al., 2022; Jutte et al., 2021). Similarly, there is a clear gap in providing adequate pediatric mental health care nationwide, with approximately 1-in-4 children receiving adequate mental health services despite the ever-increasing need and trajectory to impact lifetime health outcomes (Anderson et al., 2014; Barican et al., 2022). Therefore, we assessed patient-specific factors as predictors to connecting with mental health services to identify at-risk groups who may require additional navigation support.

### Results

Overall, 62.7% of children within PSS connected to mental health services following referral from their primary care provider from September 2017 through March 2023. Children referred to PSS for mental health service connection were primarily referred due to patient-level mental, behavioral, and developmental concerns (73.63%), followed by parent and family-level concerns (23.14%) and high-risk social needs (3.23%). Over one-third (38.7%) of the sample population were between ages 6 and 11 and a majority identified as non-Hispanic White (68.4%) and were primarily insured by Medicaid (54.1%). Significant differences in connection to mental health services were found between age groups and by SDOH opportunity level within the Social and Economic and Education domains (*p* < 0.001; Table 1). Children referred to PSS in this sample demonstrated improved connection to mental health services from 0 to < 3 years to 6 to 11 years, and then remained relatively stable through 18 years old (*p* < 0.001). Across the Social and Economic and Education domain, children living in zip codes with “very high” and “low” SDOH positions demonstrated the highest proportion of connection to services (Very High: 66.03 and 65.05%, Low: 65.23 and 67.33%), with those living in zip codes of “moderate” SDOH positions having the lowest proportion (59.24 and 60.49%, respectively).

The SEM indicated an acceptable model fit [${x}^{2}$(20) = 107.45, *p* < 0.001, TLI = 0.967, CFI = 0.984, RMSEA = 0.033 (90% CI: 0.027, 0.040)] (Table 2).

**Table 2:** Strengths and significance of effects on social drivers of health and connection to mental health services among patients triaged through Pediatric Support Services’ since inception (N = 3930)

Predictor variable	Dependent variable	β	SE	*p-value*
Child age	SDOH	.000	.020	.995
Race/ethnicity				
Non-Hispanic Black	SDOH	− .057	.106	.588
Hispanic	SDOH	− .015	.051	.776
Other	SDOH	.033	.054	.536
Insurance coverage				
Private	SDOH	.301	.035	< .001
Self-Pay/Other	SDOH	.334	.066	< .001
Sex (Ref: Male)	SDOH	.032	0.033	.342
Number of identified needs at time of referral	SDOH	− .090	.024	< .001
Parental mental health as a primary need	SDOH	− .155	.122	.205
Child age	Connection to services	.053	.010	< .001
Race/ethnicity				
Non-Hispanic Black	Connection to services	− .009	.045	.839
Hispanic	Connection to services	.004	.023	.859
Other	Connection to services	.048	.023	.036
Insurance coverage				
Private	Connection to services	− .025	.017	.137
Self-Pay/Other	Connection to services	− .059	.033	.072
Sex (Ref: Male)	Connection to services	.006	.016	.723
Number of identified needs at time of referral	Connection to services	.034	.011	.002
Parental mental health as a primary need	Connection to services	− .114	.055	.039
SDOH	Connection to services	.016	.008	.040

“SDOH” refers to the 5-point scale of COI 2.0, as described in the *Methods,* ranging from “very low” to “very high” SDOH position

Significant predictors (p < .05) are bolded

#### Factors Associated with SDOH Position

Regarding SDOH, children with private (β = 0.301, *SE* = 0.035, *p* < 0.001) or self-pay and other forms of insurance (β = 0.334, *SE* = 0.066, *p* < 0.001) are associated with a slightly higher SDOH position within their community environment, compared to children with public insurance. Further, as a child’s number of needs identified at time of referral increases, their SDOH position decreases (β = − 0.090, *SE* = 0.024, *p* < 0.001). A child’s age (β = 0.000, *SE* = 0.020, *p* = 0.995), race and ethnicity (NH Black: β = − 0.057, *SE* = 0.106, *p* = 0.588; Hispanic: β = − 0.015, *SE* = 0.051, *p* = 0.776; Other: β = 0.033, *SE* = 0.054, *p* = 0.536), sex (β = 0.032, *SE* = 0.033, *p* = 0.342), and parental mental health (β = − 0.155, *SE* = 0.122, *p* = 0.205) are not significantly associated with their SDOH position in this model.

#### Predictors of Connection to Mental Health Services

Regardless of SDOH, as children get older, they are more likely to connect with mental health services (β = 0.053, *SE* = 0.010, *p* < 0.001). Among children who identify as non-Hispanic Black or Hispanic, race and ethnicity did not affect their likelihood of connecting with mental health services (β = − 0.009, *SE* = 0.045, *p* = 0.839 and β = 0.004, *SE* = 0.023, *p* = 0.859, respectively). However, children who identify with other racial and ethnic groups—including those who identify as Asian, Multiracial, and Native Hawaiian or other Pacific Islander—are more likely to connect with mental health services than children who identify as non-Hispanic White (β = 0.048, *SE* = 0.023, *p* = 0.036), while controlling for all other factors. As a child’s number of needs at time of referral increases, their likelihood of connecting with mental health services increases (β = 0.034, *SE* = 0.011, *p* = 0.002). However, if the child is referred and their parents’ mental health is a primary reason for referral, they are less likely to connect with mental health services, compared to children whose parents do not receive mental health referrals (β = − 0.114, *SE* = 0.055, *p* = 0.039). Insurance predicts SDOH environment but does not have a significant effect on connection to mental health services (Private: β = − 0.025, *SE* = 0.017, *p* = 0.137 and Self-Pay/Other: β = − 0.059, *SE* = 0.033, *p* = 0.072). Still, children who live in communities with higher SDOH positions are more likely to connect with mental health services (β = 0.016, *SE* = 0.008, *p* = 0.040), suggesting these children have increased access to necessary resources to connect to referred services and may experience less barriers to mental healthcare than children living in low-income communities.

### Discussion

#### The Importance of SDOH in Pediatric Mental Health Care

**Fig. 2:**
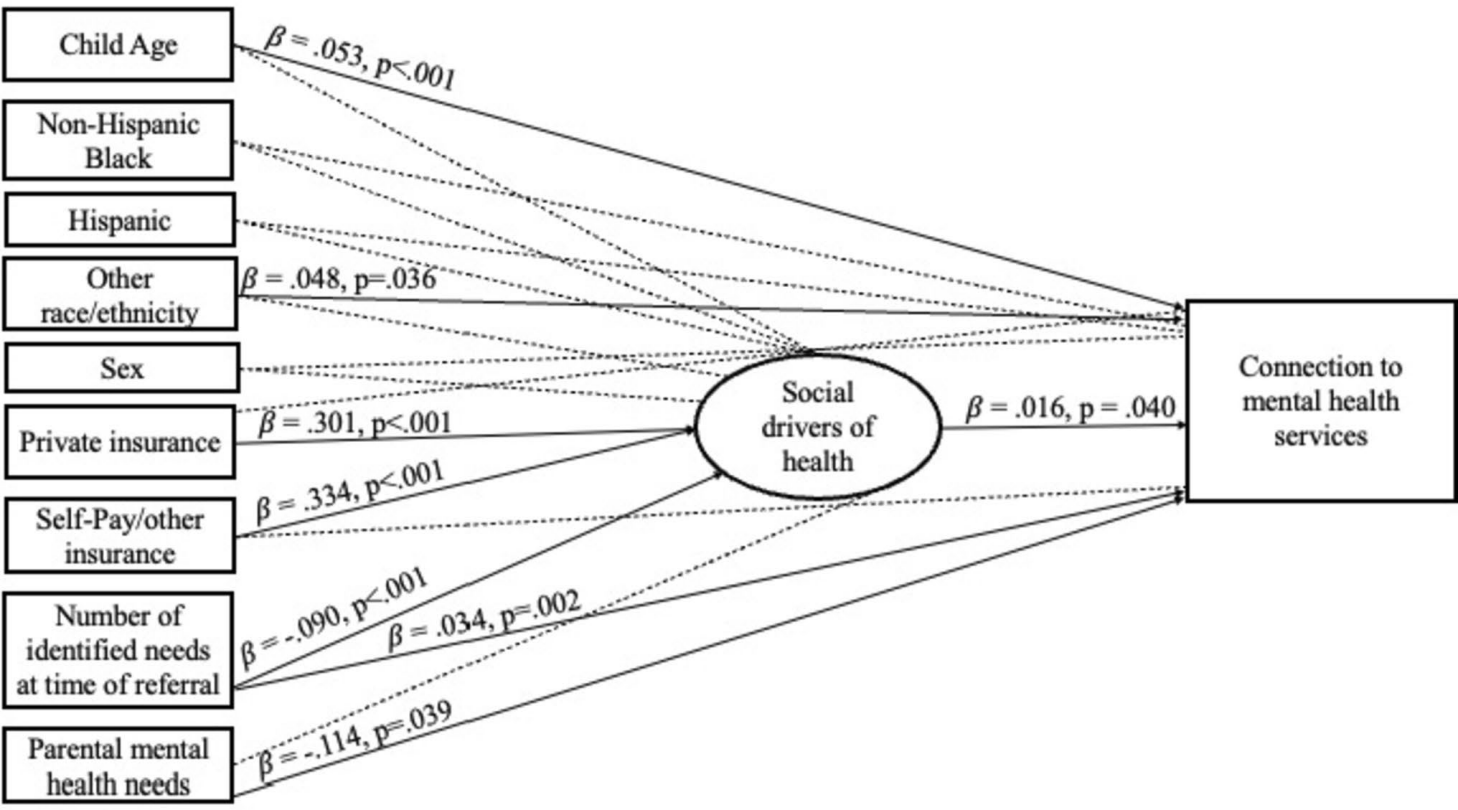
SEM of patient-and community-level factors with connection to mental health services among children triaged through PSS (from September 2017 through March 2023). β indicates standardized regression coefficient. Dashed lines indicate non-significant results (p  > .05)

Existing literature states that children living in low-income households are more susceptible to developing mental health conditions that will persist across the lifespan, and only approximately 1-in-4 children with mental health needs have access to adequate mental health services nationwide (Anderson et al., 2014; Barican et al., 2022). Children living in areas with lower SDOH estimates, often experience additional barriers to accessing appropriate care, including transportation, financial cost, availability of appointments and specialists (Anderson et al., 2014; Barican et al., 2022; Jutte et al., 2021). This study confirms these findings within our sample population as well (Fig. 2). Existing research supports risk tiering and pediatric care coordination among children based on community-and household-level factors; however, such research is limited (Swann-Thomsen et al., 2021).

Therefore, it is critical for providers to understand the context of the SDOH within the child’s household and help connect them with appropriate resources to access necessary care (Fusar-Poli, 2019; Hodgkinson et al., 2017; Matandika et al., 2022). Recent literature emphasizes that screening for SDOH is ineffective without a supportive intervention to facilitate service connection (Dworkin & Garg, 2019; Garg & Dworkin, 2016). Specific to this study, the health system in which PSS is situated within is in the process of adopting universal screeners for SDOH across primary care and has adopted the Survey of Well-being in Youth and Children (SWYC), with SDOH components among children aged 2 months to 5 years, systemwide (Berger-Jenkins et al., 2019). Children with identified SDOH needs, including household food insecurity, are referred by their primary care providers to PSS, where the referral is triaged and navigators can connect them with community-based resources. This referral process is designed for optimal carryover from referral to service connection, with the support of navigators (Garg & Dworkin, 2016).

In light of this study’s findings, navigators may provide more tailored follow-up to pediatric populations who are younger, have minimal identified referral needs, or who experience more SDOH barriers, as these groups demonstrate increased risk of not connecting with necessary mental health services. Internally, zip-code level SDOH estimates may be integrated into navigators’ referral-to-service connection recommendations, flagging youth living in comparatively higher risk zip codes or with additional challenges related to food and/or housing instability and offering additional support to overcome potential SDOH barriers to care (Dworkin & Garg, 2019; Nayak et al., 2022). With time, stratification of patient- and community-level risk factors may inform providers’ treatment plans to meet patients’ mental health needs (Burrell et al., 2023; Nayak et al., 2022).This care model is vital to pediatric comprehensive health and development, preventing long-term health conditions through early detection and treatment beyond patient’s mental health symptomology.

Further, there is limited understanding of current, widely validated navigation protocols and best practices beyond pilot studies (Kelly et al., 2019; Ustjanauskas et al., 2016). Standardization of navigation protocols to address specific patient needs, based on existing research, may improve patient outcomes while also enhancing standardization of evaluations across navigation systems (Ali-Faisal et al., 2017). Within this study’s health system, standardized training and protocols for navigators will inherently promote closed communication to inform navigator efforts and protocols, optimizing referral-to-service connection and accounting for potential SDOH barriers (Ali-Faisal et al., 2017). For instance, patients who experience housing instability may particularly benefit from PSS and its implications for mental health and navigating community-based resources(Nayak et al., 2022). Lastly, findings within this health system and from other PNPs, using standardized practices and protocols, will contribute to the generalizability of program components to promote equitable mental healthcare access across pediatric populations.

#### Adaptations to PSS

While navigators are vital to the effectiveness of PSS and similar PNPs, program adaptations must enhance navigator capacity to provide ongoing support to at-risk populations—particularly within large health systems. Within PSS, navigators use REDCap as an integrated database, merging referral data and electronic health records, to streamline data collection, reduce manual data entry error, and manage patient referrals (Harris et al., 2009; Shalhout et al., 2022). Still, current practices reporting navigator data and related referral follow-up back to primary care clinics is limited. Using emerging technology, a closed-loop system may be implemented to communicate referral information back to providers for a naturally embedded opportunity for follow-up at well-child visits systemwide (Torous et al., 2021). Additionally, this system provides an opportunity to communicate referral status back to the navigators, so they can better serve patients and their families. Closed loop referral systems may reduce opportunities for miscommunication between providers, navigators, and specialists as well as fragmented care experiences among patients and their families. This technological advancement is not intended to replace navigator personnel support, but to enhance their capacity, capitalize on existing SDOH screening practices, build efficiencies in navigator workflow to optimize PNP reach, and prepare for systemwide replication (Deferio et al., 2019; Dworkin & Garg, 2019; Torous et al., 2021).

#### Limitations

While the REDCap process is used to reduce data entry errors, this dataset is partially reliant on manual data entry. Future processes involving automation and technology innovations, such as closed loop referral systems and enhanced technological capabilities within existing software, are intended to address these limitations for future research, to improve patient outcomes, and ongoing evaluation (Torous et al., 2021). In addition, patients who are “pending” at time of initial follow-up in this study are considered “connected to services.” As part of a larger ongoing study, this research team is conducting follow-up calls with patients and families who are pending services to collect additional information related to potential barriers and duration of “pending” period will inform ongoing and future research. This information will inform the standardization of navigator protocols and ongoing navigator efforts.

Because COI 2.0 is publicly available, SDOH estimates were provided as domain scores rather than raw scores by indicator to be calculated by the research team. As a result, several indicators are reverse coded at the zip-code level, which may cause undetected and/or unintentional methodological challenges in quantitative analyses and interpretation (Appendix Table 3) (Trimmer, 2014). As such, additional research is needed to test the generalizability of the study findings relationships across contexts and datasets.

## Conclusion

Within PSS, this study found that patients who are aged birth through 3 years old, who identify with minority racial and ethnic groups, and who have limited referral needs are less likely to connect with referred mental health services. Further, those with less “opportunity” or a lower SDOH position, as measured by COI 2.0, may experience less mental healthcare access, compared to children whose families have more educational and socioeconomic resources. PSS may be adapted to involve risk tiering and providing equitable support to at-risk groups, identified through patient- and community-level factors, to address existing disparities.

## Appendix

See Table 3

**Table 3:** Childhood Opportunity Index 2.0 Indicators by Domain (Acevedo-Garcia et al., 2020)

Subdomain	Indicator	Operationalization
Domain: education		
Early childhood Education (ECE)	ECE Centers	Number of ECE centers within a 5-mile radius
	High-quality ECE centers	Number of NAEYC accredited centers within a 5-mile radius
	ECE enrollment	Percentage of 3- to 4- year olds enrolled in nursery school, preschool or kindergarten
Elementary education	Third grade reading proficiency	Percentage of third graders scoring proficient on standardized reading tests, converted to NAEP scale score points
	Third grade math proficiency	Percentage of third graders scoring proficient on standardized math tests, converted to NAEP scale score points
Secondary and postsecondary education	High school graduation rate	Percentage of ninth graders graduating from high school on time
	Advance Placement (AP) course enrollment	Ratio of students enrolled in at least one AP course to the number of 11th and 12th graders
	College enrollment in nearby institutions	Percentage of 18–24 year olds enrolled in college within a 25-mile radius
Educational and social resources	School poverty	Percentage of students in elementary schools eligible for free or reduce-price lunches
	Teacher experience	Percentage of teachers in their first and second year
	Adult educational attainment	Percentage of adults aged 25 and over with a college degree or higher
Domain: health and environment		
Healthy environments	Access to healthy food	Percentage of households without a car located further than a half-mile from the nearest supermarket
	Access to green space	Percentage of impenetrable surface areas such as rooftops, roads, or parking lots
	Walkability	EPA Walkability Index
	Housing vacancy rates	Percentage of housing units that are vacant
Toxic exposures	Hazardous waste dump sites	Average number of Superfund sites within a 2-mile radius
	Industrial pollutants in air, water, or soil	Index of toxic chemicals released by industrial facilities
	Airborne microparticles	Mean estimated microparticle concentration (PM2.5; micrograms per cubic meter)
	Ozone concentration	Mean estimated 8-h average ozone concentration (parts per billion)
	Extreme heat exposure	Number of summer days with maximum temperature above 90 degrees Farhenheit
Health resources	Health insurance coverage	Percentage of individuals aged 0–64 with health insurance coverage
Domain: social and economic		
Economic opportunities	Employment rate	Percentage of adults aged 25–54 who are employed
	Commute duration	Percentage of workers commuting more than one hour one way
Economic and social resources	Poverty rate	Percentage of individuals living in households with incomes below 100% of the federal poverty threshold
	Public assistance rate	Percentage of households receiving cash public assistance or Food Stamps/Supplemental Nutrition Assistance Program
	Homeownership rate	Percentage of owner-occupied housing units
	High-skill employment	Percentage of individuals aged 16 or older who are employed in management, business, financial, computer, engineering, science, education, legal, community service, healthcare practitioner, health technology, arts and media occupations
	Median household income	Median income of all households
	Single-headed households	Percentage of family households that are single-parent headed

Identifies indicators that are reverse coded, so higher values represent higher opportunity

## Abbreviations

PNPs: Patient Navigation Programs
PSS: Pediatric Support Services
